# Specialist PrE-hospital rEDirection for ischaemic stroke thrombectomY (SPEEDY): study protocol for a cluster randomised controlled trial with included health economic and process evaluations

**DOI:** 10.1136/bmjopen-2025-112545

**Published:** 2026-01-13

**Authors:** Lisa Shaw, Michael Allen, Jo Day, Gary A Ford, Martin James, Graham McClelland, Peter McMeekin, Helen Mossop, Catherine J Pope, Rosemary L Simmonds, Phil White, Nina Wilson, Christopher I Price

**Affiliations:** 1 Stroke Research Group, Population Health Sciences Institute, Newcastle University, Newcastle upon Tyne, UK; 2 Health and Community Sciences, University of Exeter, Exeter, UK; 3 Oxford University Hospitals NHS Foundation Trust, Oxford, UK; 4 Medical Sciences Division, University of Oxford, Oxford, UK; 5 Royal Devon University Healthcare NHS Foundation Trust, Exeter, UK; 6 Department of Nursing, Midwifery & Health, Northumbria University, Newcastle upon Tyne, UK; 7 Biostatistics Research Group, Population Health Sciences Institute, Newcastle University, Newcastle upon Tyne, UK; 8 Nuffield Department of Primary Care Health Sciences, University of Oxford, Oxford, UK; 9 Stroke Research Group, Translational and Clinical Research Institute, Newcastle University, Newcastle upon Tyne, UK

**Keywords:** STROKE MEDICINE, Randomized Controlled Trial, Treatment Outcome

## Abstract

**Background:**

Outcome from large vessel occlusion stroke can be significantly improved by time-critical thrombectomy but treatment is only available in regional comprehensive stroke centres (CSCs). Many patients are first admitted to a local primary stroke centre (PSC) and require transfer to a CSC, which delays treatment and decreases the chance of a good outcome. Access to thrombectomy might be improved if eligible patients could be identified in the prehospital setting and selectively redirected to a CSC. This study is evaluating a new specialist prehospital redirection pathway intended to facilitate access to thrombectomy.

**Methods and analysis:**

This study is a multicentre cluster randomised controlled trial with included health economic and process evaluations. Clusters are ambulance stations (or teams) which are work bases for ambulance practitioners. Intervention allocated ambulance practitioners use the Specialist PrE-hospital rEDirection for ischaemic stroke thrombectomY (‘SPEEDY’) pathway which comprises initiation according to specific criteria followed by contact with CSC staff who undertake a remote assessment to select patients for direct CSC admission. Control allocated ambulance practitioners continue to provide standard care which comprises admission to a local PSC and transfer to a CSC for thrombectomy if required. A co-primary outcome of thrombectomy treatment rate and time from stroke symptom onset to thrombectomy treatment will evaluate the impact of the pathway. Secondary outcomes include key aspects of emergency care including prehospital/hospital time intervals, receipt of other treatments including thrombolysis, and performance characteristics of the pathway. A broad population of all ambulance practitioner suspected and confirmed stroke patients across participating regions is being enrolled with a consent waiver. Data about SPEEDY pathway delivery are captured onto a study case record form, but all other data are obtained from routine healthcare records. Powered on a ‘primary analysis population’ (ischaemic stroke patients with pathway initiation criteria), 894 participants will detect an 8.4% difference in rate and data from 564 thrombectomy procedures will detect a 30 minute difference in time to treatment. The full study population is estimated to be approximately 80 000. Regression modelling will be used to examine primary and secondary outcomes in several analysis populations. The economic analyses will include cost-effectiveness and cost–utility analyses, and calculation of willingness to pay at a range of accepted threshold values. The process evaluation involves semi-structured interviews with professionals and patient/family members to explore views and experiences about the SPEEDY pathway.

**Ethics and dissemination:**

This study has ethical, Health Research Authority and participating NHS Trust approvals.

Dissemination of study results will include presentations at national and international conferences and events, publication in peer-reviewed journals, and plain English summaries for patient/public engagement activities.

**Trial registration number:**

ISRCTN77453332.

**STRENGTHS AND LIMITATIONS OF THIS STUDY**

- This is an appropriately powered cluster randomised controlled trial to evaluate the impact of a new specialist prehospital redirection pathway intended to facilitate access to stroke thrombectomy treatment.
- Due to the nature of the intervention, cluster randomisation is appropriate to minimise contamination between groups.
- Participant enrolment is via a consent waiver to avoid a population bias.
- Enrolled participants comprise all ambulance suspected and confirmed stroke across participating regions to allow the impact of the pathway to be assessed for different populations.
- Data are being obtained from routine healthcare records which improves efficiency, but this may also raise concerns about data quality.

## Background

 Stroke is the most common cause of severe adult disability,^1 2^ but outcomes can be significantly improved for selected patients by time-critical treatments.^3^,^6^ To provide these rapidly and achieve better outcomes, ambulance practitioners convey patients with suspected stroke to the nearest stroke unit (also called ‘primary stroke centre’ (PSC)) for specialist clinical assessment.^7^ Here, eligible patients with ischaemic stroke are treated using intravenous thrombolysis to dissolve any thrombus reducing cerebral blood flow.^7 8^ This therapy is routinely provided in all PSCs and avoids severe disability for one in seven treated.^4^

For ischaemic stroke which is caused by occlusion of a large blood vessel (large vessel occlusion (LVO)), treatment with mechanical thrombectomy, with or without intravenous thrombolysis, results in better outcomes.^5 6^ Considering patients who are known to be within 6 hours of symptom onset, thrombectomy provides a significantly better chance of recovering independence in daily activities compared with thrombolysis alone (adjusted OR 2.49 (95% CI 1.76 to 3.53); number needed to treat 2.6).^5^ However, a favourable outcome is strongly time dependent and for every 60 minutes that thrombectomy is delayed, there is an average 6.7% absolute reduction in good outcome.^5^ As thrombectomy is not a risk-free procedure and carries a 3% absolute risk of significant neurological deterioration, later treatment for this group is not only futile but also potentially harmful.^5 6^

Although all PSCs provide thrombolysis, thrombectomy is only available in regional ‘comprehensive stroke centres’ (CSCs) because the procedure requires experienced interventionists operating in highly specialised facilities. For patients who live outside of a CSC catchment area, a thrombectomy suitability assessment and initiation of thrombolysis (if eligible) is undertaken at a local PSC, before secondary transfer by ambulance to a CSC.^9 10^ This thrombectomy assessment, initiation of thrombolysis and secondary transfer creates a median (IQR) delay of 100 (85, 129) minutes before the thrombectomy procedure starts,^11^ thereby reducing treatment effectiveness whilst creating additional local resource demands. A systematic review consisting of non-randomised comparisons of data from patients admitted directly to thrombectomy providers versus secondary transfer showed fewer good functional outcomes among transferred patients (OR 0.87 (95% CI 0.81 to 0.93)).^12^ In England, it is estimated that approximately 70% of people live in areas where access to thrombectomy requires a secondary transfer for treatment,^13^ and as such experience a significant delay relative to those living near to a CSC. Furthermore, it has also been demonstrated that National Health Service (NHS) patients who are not directly admitted to a CSC have a significantly lower chance of receiving thrombectomy.^14^

Whilst one solution to the thrombectomy access issue is to transport all ambulance suspected stroke patients directly to a CSC, this is not feasible due to limited capacity.^10^ Furthermore, such a model would also result in many patients being displaced from a local unit when not eligible for thrombectomy. Up to 40% of people suspected to be stroke by ambulance practitioners receive a non-stroke mimic diagnosis after hospital assessment^15^ and within the remaining stroke population, only some have LVO stroke eligible for thrombectomy. Instead, an alternative option is to identify people with LVO stroke who are potentially suitable for thrombectomy in the prehospital ambulance setting and selectively redirect to a CSC, rather than initial admission to the nearest PSC.

Selective redirection models have begun to be evaluated and to date there are two published randomised controlled trials (RCTs). In the RACECAT trial^16 17^ (Catalonia, Spain), which reported in 2022, paramedics used the Rapid Arterial oCclusion Evaluation (RACE) scale^18^ as the means of LVO stroke identification and patients scoring greater than 4 (out of 9) points were randomised to redirection to a thrombectomy provider or local admission to the nearest stroke unit. Patients in the intervention group were more likely to receive thrombectomy (OR 1.46 (95% CI 1.13 to 1.89)) and there was a 56 minute reduction in time to treatment. However, thrombolysis treatment was less likely (OR 0.59 (95% CI 0.45 to 0.70)) and slower (34.5 minutes). Overall, there was no effect on patient functional status at day 90 after stroke (aOR 1.03 (95% CI 0.82 to 1.29)). Of the 679 patients included in the redirection (intervention) group, 37% (n=253) received a thrombectomy, therefore 63% of patients were redirected and did not receive thrombectomy treatment. In a secondary analysis published in 2023, concern was raised about poorer outcomes for redirected haemorrhagic stroke patients (141/679 (20.8%)).^19^

The second redirection trial^20^ (conducted in Denmark) which reported in 2023, only recruited 171 of 600 planned patients due to a slow enrolment rate. This study involved a two-step patient assessment which considered both LVO identification and thrombectomy suitability prior to randomisation. Ambulance practitioners firstly used the Prehospital Acute Stroke Severity (PASS) scale, and if patients scored above a threshold (2 or more), this triggered a telephone call to a regional thrombectomy centre. The call taker then determined whether the patient could be a possible thrombectomy candidate, and if so, the patient was randomised to redirection to the CSC or usual care admission to the nearest PSC. Compared with standard care, more of the intervention group received thrombectomy (63% vs 53%), with a reduction in onset to treatment time of 35 minutes (p=0.007). More intervention patients also received thrombolysis (78% vs 67%), but with a delay of 30 minutes (p=0012). Overall, no difference was found in patient functional outcome at day 90 (aOR 1.42 (95% CI 0.72 to 2.82)). Of the 87 patients included in the redirection (intervention) group, 33% (n=29) received a thrombectomy and therefore 67% were redirected without treatment.

A two-stage process of ambulance practitioner clinical scale assessment triggering a CSC remote review has also been described during a non-randomised observational study of redirection for thrombectomy in Stockholm (published 2020).^21^ Ambulance nurses who suspected stroke based on assessment using the Face Arm Speech Test (FAST) further graded arm and leg strength, and if moderate or severe weakness was present, a thrombectomy centre stroke specialist was consulted by telephone. The call taker assessed likely suitability for thrombectomy and made a decision about whether to convey directly to the thrombectomy centre or to the nearest local stroke centre. Compared with historical controls from the year before, the proportion of patients undergoing thrombectomy after direct admission increased from 24% to 73% (p<0.001), while median onset to thrombectomy time reduced from 206 (IQR 160–280) to 137 minutes (IQR 118–180) (p<0.001). Thrombolysis timings compared with historical controls were unchanged. For 25% of patients, thrombectomy was still accessed through secondary transfer from a PSC, either because they were not initially thought to be suitable during remote prehospital assessment, or because the assessment was not activated due to failure to identify that the patient’s symptoms were possibly due to stroke. Overall, of the patients directly admitted to the CSC, 26% received a thrombectomy and performance characteristics of the assessment process were accuracy of 87% for LVO stroke identification and specificity of 91% for receipt of thrombectomy.

During the time that the above-described studies were ongoing, funding was secured to develop and evaluate a selective redirection model for the UK. Qualitative work during 2021–2022^22^ established the acceptability of a new prehospital pathway which considered the need for accurate identification of patients with LVO stroke and suitability for thrombectomy, as well as the ability for implementation into existing healthcare infrastructure. The pathway comprises the following steps:

1. *Initiation* by any grade of ambulance practitioner using information routinely collected during suspected stroke assessment which indicates LVO may be likely.
2. *Enhanced prenotification* directly with the CSC instead of routine prenotification to the nearest local PSC.
3. *A remote specialist assessmen*t undertaken by a member of the CSC to make a decision about whether the patient should be redirected to the CSC or continue to the local PSC.

This pathway intends to provide firstly high sensitivity for LVO stroke identification and then secondly high specificity for thrombectomy suitability, with the aim of ensuring that the most appropriate patients are conveyed directly to a CSC, ie, keeping non-LVO patients to a minimum. By using information already routinely collected during suspected stroke assessment and a remote assessment by CSC staff, the pathway should be easily deployed by both ambulance services and CSC.

The effectiveness of this pathway is now being assessed in an ongoing large cluster RCT with included health economic and process evaluations. This trial will be an important contribution to the recent literature about selective redirection and also responds to the conclusions of the two previous trials which call for further studies to answer the question about this approach to access thrombectomy. This manuscript describes the protocol for the ongoing study.

## Methods and analysis

### Study aim

To determine the clinical and cost effectiveness of a novel specialist prehospital redirection pathway intended to facilitate thrombectomy treatment for acute stroke.

### Study objectives

- To determine the impact of the novel specialist prehospital redirection pathway upon thrombectomy rate and time to treatment (co-primary outcome).
- To determine the impact of the novel specialist prehospital redirection pathway upon key aspects of emergency care including time intervals from emergency call and hospital admission to first brain imaging and thrombolysis treatment (if given).
- To determine the impact of the novel specialist prehospital redirection pathway upon direct admission and transfer rates to CSCs.
- To report the performance characteristics (eg, sensitivity and specificity) of the novel specialist prehospital redirection pathway for identification of patients who receive thrombectomy.
- To determine the cost-effectiveness of the novel specialist prehospital redirection pathway relative to standard NHS care.
- To report professional and patient/family member views and experiences about the novel specialist prehospital redirection pathway (process evaluation).

### Study design

The study is a multicentre cluster RCT with included health economic analysis and process evaluation. Clusters are ambulance stations (work bases for ambulance practitioners) or teams within regional ambulance services. Stations/teams are randomised to intervention (ie, provision of the specialist prehospital redirection thrombectomy pathway) or standard care (ie, local PSC admission with subsequent secondary transfer for thrombectomy following local assessment). A cluster design was chosen to minimise contamination between study groups and to avoid delays to care processes which could occur if individual patient randomisation were utilised.

### Study setting

The study is taking place across selected NHS regional ambulance services, PSCs and CSCs in England. To be considered for inclusion, ambulance services had to cover a geographical region where the stroke service configuration was such that a large proportion of the population accessed thrombectomy via secondary transfer. In addition, the ambulance service in conjunction with the regional CSC had to be willing to deploy the specialist prehospital redirection pathway.

### Cluster randomisation

Within each ambulance service region, stations/teams were eligible for randomisation if the ambulance practitioners attend incidents in geographical areas where suspected stroke patients are usually conveyed to a PSC which makes thrombectomy referrals to the participating CSC. In addition, stations/teams which usually convey directly to the participating CSC were also randomised so that all stations/teams across a region were included. This choice of all stations/teams (ambulance practitioners) across a region was because (1) ambulance practitioners and vehicles are often moved around according to operational service demand and can serve both PSCs and CSCs irrespective of station base/team, hence this approach ensures that all pathway eligible patients will be attended by a randomised ambulance practitioner and (2) the full study population can be identified (see also Study population, below).

Stations within an ambulance service region were only excluded from randomisation if they had a non-routine function and staff would not be deployed to suspected stroke incidents (eg, the station is the base for Hazardous Area Response Team practitioners only), or, if they were in geographical regions where it would be very unlikely that attended patients would be conveyed to a study relevant PSC or CSC. For example, some ambulance service regions serve more than one CSC and staff from specific stations were highly likely to only attend incidents where conveyance was to a PSC which refers to the non-study CSC.

Within each participating region, included stations/teams were allocated 1:1 between intervention and standard care using covariate constrained randomisation,^23^ balanced according to size (defined as total number of staff) and distance from the CSC. Balancing on these variables was to ensure that intervention and standard pathway ambulance practitioners were approximately equally matched in terms of important operational characteristics. For participating regions with more than one included CSC, randomisation was stratified by centre, or centre was used as a covariate in the balance metric, depending on the number of ambulance stations involved.

### Study intervention

The specialist prehospital redirection pathway (SPEEDY pathway) consists of:

1. *Initiation* by any grade of ambulance practitioner using information routinely collected during suspected stroke assessment.

Intervention ambulance practitioners initiate the pathway if:

- the incident is within a location where routine conveyance is to a PSC which refers to a participating CSC (ie, secondary transfer area).
- the incident is within the time window agreed by the relevant participating CSC for operating the SPEEDY pathway (eg, 8:00 to 20:00).
- the standard ambulance practitioner assessment indicates the following clinical criteria:
  - a clinical impression of suspected acute stroke with two symptoms of the standard FAST test both present: arm weakness and speech disturbance.
  - the symptoms began within the last 5 hours, or it is within 5 hours since waking with these symptoms.
  - a low risk of an endangered airway that would require additional urgent medical attention (Alert, Vocal response or Pain response (but not Unresponsive) on the AVPU scale).

When two symptoms of the standard FAST are present sensitivity for LVO is 95% among stroke patients treated with intravenous thrombolysis (n=3505)^24^ and 95% among all ischaemic stroke admissions (n=1006).^25^ This high sensitivity is desirable for pathway initiation and using the standard FAST test negates a need for additional ambulance practitioner training. The commonest FAST symptoms are arm weakness (87% patients) and speech disturbance (72% patients). These also demonstrate the greatest agreement between ambulance and hospital practitioners during assessment (kappa=0.77 (95% CI 0.55 to 0.99) and 0.69 (95% CI 0.56 to 0.82), respectively), whereas facial weakness is less frequent (62% patients) and there is much lower agreement (kappa=0.49 (95% CI 0.36 to 0.62)).^26^ Therefore, to optimise performance, arm weakness and speech disturbance were the two FAST symptoms chosen.
The time-critical nature of thrombectomy treatment also means an upper time boundary for pathway initiation is necessary. This is specified as within 5 hours of symptom onset or within 5 hours of waking with symptoms to allow treatment to be performed within a timeframe supported by the strongest existing evidence. Although more recent clinical trials have reported that thrombectomy is effective up to 24 hours since patients are last known to be symptom free,^27^ selection of such cases for treatment requires advanced brain imaging which is not yet part of standard care in some PSCs. The pathway was also designed such that it fitted alongside existing standard care.

1. *Enhanced prenotification* directly with the CSC instead of routine prenotification to the nearest local PSC.

Previous clinical studies have shown that ambulance prenotification during transportation to the admitting hospital reduces the time between arrival and thrombolysis administration^28 29^ and is recommended in National Clinical Guidelines.^7^ The pathway involves *enhancement of standard pre-notification* where ambulance practitioners will exchange additional information with a remote specialist assessor. This is necessary to support the pathway redirection decision, but providing details to the thrombectomy centre team earlier will also facilitate processes following patient arrival, for example, alerting the thrombectomy treatment suite staff to prepare. In each participating region, a dedicated telephone number is provided for ambulance practitioners to access the CSC remote assessors.

1. *A remote specialist assessment* undertaken by a member of the CSC team to make a decision about whether the patient should be redirected to the CSC or continue to the local PSC.

This assessment involves initial use of a checklist to confirm pathway eligibility (ie, routine conveyance would be to a PSC, FAST results, symptom onset time, AVPU score) and record additional clinical characteristics including key vital signs required to inform a decision about the likely appropriateness of thrombectomy. Thereafter, CSC staff will seek extra information as required which may influence the destination choice. A decision about direct admission to the CSC or to continue to the local PSC is subsequently made.

#### Other information about the pathway

As any suspected stroke requires urgent assessment in relation to time critical treatments, intervention ambulance practitioners are advised to revert to standard care (ie, admission to the local PSC) if they have been unable to make contact with the remote assessor after two attempts at a telephone call. Similarly, the remote assessment should not exceed 10 minutes in duration, and if this occurs without a likely imminent destination decision (eg, CSC staff need additional advice which is not immediately accessible), both ambulance practitioners and CSC staff have been advised to terminate the discussion and revert to local PSC admission.

Patients who are eligible for pathway deployment but this is not undertaken (eg, due to failure of ambulance initiation), or who have a remote assessment which concludes direct transfer to the CSC is not appropriate, will still undergo all usual care assessments on arrival at the local PSC and subsequently may still receive thrombectomy through the standard secondary transfer route if this is considered required. All such patients will remain assigned to the study intervention group.

### Study control (standard care)

Standard care content for ambulance assessment is defined by National Clinical Guidelines for Stroke^7^ and comprises use of a validated symptom identification checklist to identify suspected stroke (ie, FAST), confirmation of symptom onset time, recording of conscious level, exclusion of hypoglycaemia by capillary blood sampling and telephone prenotification to the nearest local stroke centre (comprehensive or primary) before arrival.

During the study timeline, there are no planned major changes for the routine prehospital processing of suspected stroke. If any region undergoes a significant change which involves redirection for possible LVO stroke, a review of study viability for that area will be undertaken.

**Figure 1:**
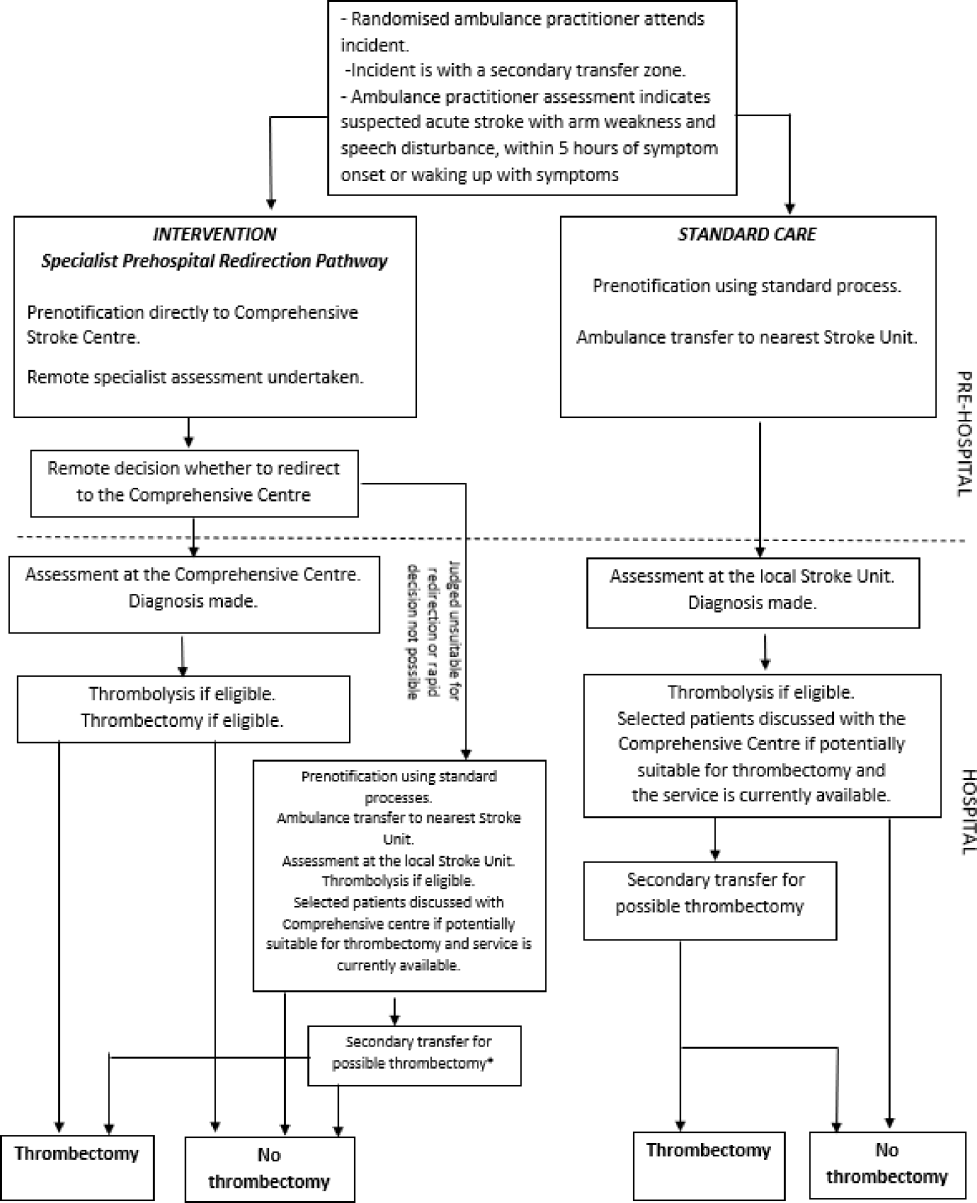
Summary of intervention and standard care access to thrombectomy treatment for the ‘target’ population. ^∗^Any intervention group patients who are not redirected will undergo usual care assessment on arrival at the local Primary Stroke Centre and may still receive thrombectomy through the standard secondary transfer route.

Figure 1 illustrates intervention and standard care access to thrombectomy treatment for the population who meet the pathway initiation criteria (also called ‘target population’, see also ‘Study population’, below).

### Implementation of the study intervention and control

#### Implementation for ambulance practitioners

Any grade of ambulance practitioner based within a randomised station/team that would normally attempt to identify suspected stroke is involved in the study.

When the study launches in each region, intervention ambulance practitioners are provided with information about the trial including pocket-sized cards covering pathway initiation criteria and specific contact details for the participating CSC team. The pathway was purposely designed to use routinely available information and as such negate a need for trial-specific training. Practitioners are instructed to start using the intervention pathway on a specific date in synchronisation with the receiving CSC.

Ambulance practitioners based within standard care stations are informed that a study about thrombectomy is taking place and routinely recorded data will be used, but otherwise instructed to continue with standard care pathways.

As double-crewed ambulances are typically dispatched to suspected stroke patients, it is expected that two ambulance practitioners will attend most incidents. Whilst typically this should be two practitioners from the same station/team and therefore the same randomisation allocation, there may be occasions when this is not the case and both an intervention and a control practitioner are in attendance. In this latter situation, the patient is considered allocated to the intervention group and the ambulance practitioners have been advised that the intervention pathway should take precedent.

During the duration of the trial, it is anticipated that the ambulance practitioner workforce within each region will be subject to change, ie, some practitioners will leave the service and new employees will commence. Because the study population comprises all suspected and confirmed strokes seen by randomised ambulance practitioners across a participating region, newly employed ambulance practitioners will become involved in the study when they commence their employment. If practitioners employed after study launch were excluded from involvement, the study population would be incomplete. New employees will be provided with details about the study in keeping with their allocated station.

It is also anticipated that some ambulance practitioners will move station bases during the trial because of regional ambulance service workforce management decisions. In order to maintain the integrity of the trial, all ambulance practitioners will be instructed that they retain their original randomisation allocation irrespective of any operational station moves, ie, retain either the group of their station at study launch or the group of their station at commencement of new employment.

#### Implementation for CSC staff

CSC stroke teams will receive study specific training about use of the intervention pathway and remote specialist assessment.

### Study population

Although by its nature the pathway is only intended for use with *some* suspected stroke patients (see also ‘Study intervention’ above), deployment of the pathway is likely to have wider impacts for stroke services across ambulance regions serving PSCs and CSCs. The study will examine outcomes within several analysis populations to report both the impact on the population eligible to receive the pathway and subsequent thrombectomy, and other populations where effects may also be seen. The overall study population comprises patients where:

- an ambulance practitioner from a randomised station/team attends the incident.
- conveyance is to a PSC which refers patients to the participating CSC, or, directly to the participating CSC (ie, the incident is in either a secondary transfer area or an area where the CSC is accessed directly).
- acute stroke is suspected by the attending ambulance practitioner (ie, FAST positive or any observed new focal neurological symptoms which indicate acute stroke according to the ambulance practitioner’s clinical judgement) OR acute stroke was diagnosed following arrival at a participating PSC or CSC irrespective of ambulance practitioner initial judgement of symptom cause.

The important ‘sub’ populations which will be used to examine the impacts of the SPEEDY pathway are as follows:

- Ambulance suspected acute stroke: ‘Ambulance suspected acute stroke population’.
- Ambulance suspected stroke demonstrating pathway clinical criteria, assessed within the agreed time window for the relevant region and usual conveyance would be to a PSC: ‘Target population’.
- Ambulance suspected stroke demonstrating pathway clinical criteria, assessed within the agreed time window for the relevant region and usual conveyance would be to a PSC, plus, following hospital assessment, a diagnosis of ischaemic stroke is assigned: ‘***Primary analysis population***’.

The population selected for the primary analysis reflects the group where direct effects of the intervention can be evaluated, ie, the population eligible for both pathway deployment and subsequent thrombectomy treatment.

**Figure 2:**
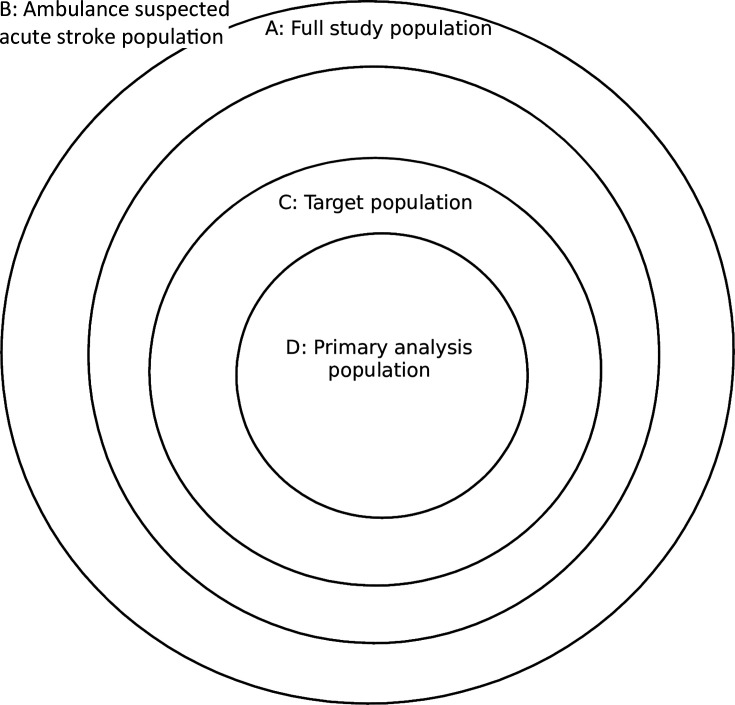
Study populations.

All populations are illustrated in figure 2.

### Participant enrolment

In keeping with previous cluster randomised health service trials,^30^ a waiver of individual patient consent was granted for participant enrolment and data collection for this trial. This approach was proposed and justified for the reasons outlined below. Note that enrolment for the process evaluation is a separate procedure and detail is provided in ‘Process evaluation’ below.

1. Thrombectomy for acute stroke is a National Institute for Health and Care Excellence (NICE) recommended treatment commissioned by NHS England, with suboptimal delivery in most regions. The study intervention does not change clinical eligibility for thrombectomy or reallocate treatment decision responsibility but aims to facilitate access to the procedure for patients that meet the established treatment criteria.
2. The study intervention pathway carries negligible risk of harm whether or not direct CSC admission is accepted or declined. Where direct CSC admission is declined, patients will still be conveyed urgently to the local PSC and undergo emergency stroke assessment. If following a PSC assessment, suitability for thrombectomy is confirmed, a secondary transfer will occur in keeping with standard care. Where direct CSC admission is accepted, the transfer will be monitored by ambulance practitioners who will respond to any changes in patient status as per usual care. If, following arrival, thrombectomy is judged not to be appropriate, the medical team will still provide all treatments required.
3. All data required for the study can be obtained from routinely recorded clinical service data; there is no burden on patients for research visits (see also ‘Data collection’ below).
4. Obtaining consent from patients who have any part of the pathway deployed, corresponding standard care patients, or the full study population would be very challenging and render the research impractical or bias:
  - Suspected stroke is a time-critical condition and introducing consent during ambulance assessment would be unacceptable due to delays to care.
  - Although consent could potentially be deferred until emergency assessments have been completed at hospital, there is no routine linkage of ambulance recorded data to hospital data to enable hospital research staff to identify eligible patients. An incomplete and bias study population would result due to failure to identify all relevant patients.
  - Deferred hospital consent is also not straightforward as effects of suspected stroke/confirmed stroke can result in a lack of mental capacity, communication difficulties and early mortality. The previous experience of the study investigator team is that complicated consent processes also result in incomplete enrolment of eligible patients and risk a bias sample. In particular, patients suitable for thrombectomy treatment, the target of the study intervention, are often the most seriously affected by stroke and consequently the most likely not to be enrolled if consent were required.
  - Each NHS stroke centre assesses approximately 5–10 suspected stroke patients per day, and the previous experience of the study investigator team is that consent processes for this volume of patients are not practical, further adding to the risk of an incomplete and bias sample.
  - Following emergency assessment at hospital, suspected stroke/confirmed stroke patients can be located in multiple different wards or indeed rapidly discharged or transferred to a different hospital site, additionally hindering attempts at consent and resulting in lack of enrolment.

### Data collection

Other than data describing delivery of the intervention pathway, all data required for analysis and reporting of the cluster trial (including health economic evaluation) are routinely collected by ambulance or hospital services. In addition, data required at the hospital level for patients with a diagnosis of stroke are provided to the Sentinel Stroke National Audit Programme (SSNAP).^11^

Three methods of data collection are being used to generate four datasets which require linkage.

Table 1 summarises the data required for the study, the routine location of these data and the data collection method. The online supplemental file lists the data items obtained in each dataset.

**Table 1:** Study data required, routine location and data collection method

Data required for the study	Routine location of data	Data collection for this study
Ambulance assessment parameters and care process information for patients suspected to be stroke by the attending ambulance practitioner and conveyed to a study CSC or feeding PSC.	Ambulance service	Data provided from participating ambulance services via regular exports from their existing electronic routine care records. Data requested will be for patients where stroke was suspected by an ambulance practitioner and the patient was conveyed to a study CSC or feeding PSC.
Hospital categorised diagnosis for patients who were suspected to be stroke by the attending ambulance practitioner and conveyed to a study CSC or feeding PSC. Diagnosis categorised as: ischaemic stroke haemorrhagic stroke non-stroke mimic condition	First admission hospital (PSC or CSC) SSNAP if a diagnosis of stroke is assigned	Data provided from SSNAP via regular exports of their dataset. Data requested will be for patients admitted by ambulance to study CSCs or feeding PSCs. SSNAP does not contain data from patients diagnosed with non-stroke mimic conditions and therefore this diagnostic category will be assumed for patients within the ambulance dataset who do not have a matching SSNAP record.
Hospital categorised diagnosis for patients who were *not *suspected to be stroke by the attending ambulance practitioner and conveyed to a study CSC or feeding PSC. Diagnosis categorised as: ischaemic stroke haemorrhagic stroke	First admission hospital (PSC or CSC) SSNAP	Data provided from SSNAP via regular exports of their dataset. Data requested will be as above.
Clinical characteristics and care received at hospital for patients with a diagnosis of stroke and conveyed to hospital by ambulance.	First admission hospital (PSC or CSC) SSNAP	Data provided from SSNAP via regular exports of their dataset. Data requested will be as above.
Thrombectomy treatment parameters for patients conveyed to hospital by ambulance (if received).	CSC	Data provided from participating CSCs via a standard research ‘case record form’. The case record form exists in paper and electronic formats (secure online study database) and data are collected contemporaneously for each patient who receives thrombectomy. All data are added to the online database either directly or from the initial paper record, according to CSC preference. This research-specific data collection form is called: ‘CSC thrombectomy treatment log’.
Study intervention pathway ‘SPEEDY pathway’ content (if received)	CSC	Data provided from participating CSCs via a standard research ‘case record form’. A paper case record form is completed contemporaneously for each call from an ambulance practitioner activating the intervention pathway. The data from the paper form is subsequently uploaded into the online study database. This research-specific data collection form is called: ‘CSC SPEEDY call log’.

CSC, comprehensive stroke centre; PSC, primary stroke centre; SPEEDY, Specialist PrE-hospital rEDirection for ischaemic stroke thrombectomY; SSNAP, Sentinel Stroke National Audit Programme.

#### Data linkage

Because of the nature of the study, some patients will NOT link across all datasets. For example, an ambulance suspected stroke case which receives a non-stroke mimic diagnosis following hospital assessment will not have a SSNAP record because SSNAP only obtains data on stroke cases. All data are required for analyses, not solely data from patients who link across every dataset.

**Figure 3:**
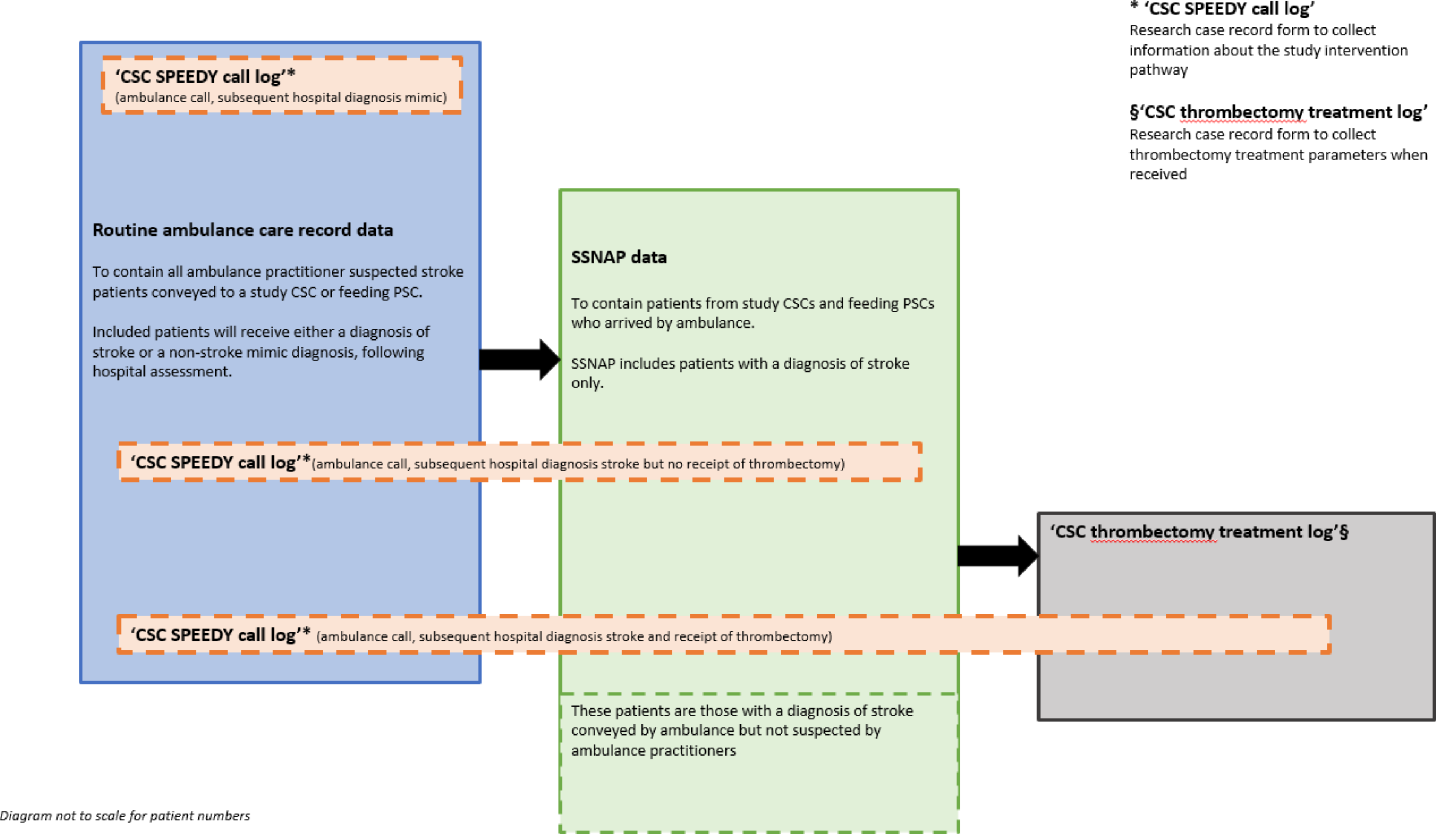
Study datasets and linkage. CSC, comprehensive stroke centres; PSC, primary stroke centre; SPEEDY, Specialist PrE-hospital rEDirection for ischaemic stroke thrombectomY; SSNAP, Sentinel Stroke National Audit Programme.

Figure 3 summarises the study datasets and linkage.

Linkage is centralised at the study co-ordinating centre at Newcastle University, England. Identifiable patient information is not sought and instead the main data items being used for linkage are:

- first admission hospital name.
- first admission hospital arrival date and time.
- month and year of date of birth (or age if date of birth is unknown).
- gender.

#### Assignment of randomisation group

All patients will be assigned to a randomisation group according to the allocation of the attending ambulance practitioner. The ambulance data exports contain information on attending practitioners and/or stations/teams such that randomisation group can be assigned. For patients in the full study population where ambulance practitioners did not suspect stroke and this was diagnosed only after arrival at hospital, information on attending practitioners (or station/team) will be sourced in arrears from the relevant ambulance service.

### Blinding

Due to the nature of the study intervention, it is not possible to blind intervention ambulance practitioners or receiving hospital stroke teams. However, because the study is cluster randomised, staff cannot influence group allocation or select patients for inclusion in the trial; all patients who meet the eligibility criteria will be included and assigned to a study group based on the randomisation allocation of the attending ambulance practitioner. Patients seen by intervention group ambulance practitioners will be included in the study whether or not the intervention pathway was deployed, and whether or not thrombectomy was received.

Participating sites are asked to conceal study group information from the clinician performing thrombectomy, which should be possible because NHS neuroradiologists are not routinely involved in urgent arrival care processes. Thrombectomy treatment decisions are based on clinical details conveyed via the hospital stroke team and brain imaging appearances. CSCs already receive patients from outside their immediate area through standard (secondary transfer) care and therefore information about a patient’s original locality would not by itself cause unblinding.

### Withdrawal and safety

Due to the nature of this cluster trial which uses a waiver of consent and data collection from routine sources, a typical trial withdrawal process is not appropriate. However, information about the study is available at NHS sites (posters, leaflets) and patients whose data may be included can request more information and removal of data should they wish.

Similarly, due to the nature of the trial, a standard safety monitoring and reporting system is not appropriate. As described above, there should be minimal risk to people who receive any part of the study intervention which aims to facilitate access to an existing evidence-based treatment. However, data collected for the study include care processes indicators (eg, time to hospital arrival and time to receipt of intravenous thrombolysis) and health outcomes which are intermittently reviewed unblinded to randomisation group by a study Data Monitoring Committee (Safety Committee).

### Outcomes

#### Primary outcome

This study has a co-primary outcome of:

1. thrombectomy rate.
2. the time from stroke symptom onset to thrombectomy (when thrombectomy is received).

The primary analysis will be conducted using the primary analysis population (see also Study population’, above).

If the stroke symptom onset time is unclear, the ‘last known well’ time will act as the onset time in keeping with standard clinical practice.

Time of thrombectomy will be defined by the time of arterial puncture whether or not thrombus is subsequently removed.

#### Secondary outcomes

1. Thrombectomy treatment rate and time to treatment (using other analysis populations).
2. Key ambulance time intervals using the following data: emergency call, ambulance dispatch, arrival on scene, leave scene, arrival at first hospital.
3. Key hospital time intervals using the following data: stroke symptom onset, hospital arrival, brain imaging, thrombolysis (when thrombolysis is received).
4. Thrombolysis rate.
5. Radiological success in re-canalising the arterial occlusion (assessed by modified TICI score).^31^
6. Stroke severity (National Institute for Health Stroke Scale (NIHSS)^32^ at 24 hours post reperfusion treatment.
7. Total length of hospital stay (stroke patients only).
8. Dependency at discharge (Modified Rankin Scale (mRS)^33^ including death) from total hospital stay (stroke patients only).
9. CSC admission and transfer rates.
10. Length of stay at a CSC (stroke patients only).

### Sample size

The study is powered using the primary analysis population (as defined above in ‘Study population’). The primary analysis population was chosen because it is the group where direct effects of the intervention can be most appropriately assessed, ie, the population eligible for both pathway deployment and subsequent thrombectomy treatment.

Considering the primary analysis population, the study is powered to detect a clinically meaningful reduction of 30 minutes in the time from stroke onset to thrombectomy and to demonstrate whether there is a change to the thrombectomy treatment rate.

Time to treatment: For 90% power (one-sided 5% significance level) to conclude that the intervention reduces the time from onset to treatment, data from *564* participants are required. This calculation assumes a random effect for clusters (ambulance stations), 1:1 allocation of 150 clusters to the two groups, and an intracluster correlation of 0.01 (based on previous similar ambulance trials which demonstrated minimal clustering).^34 35^ The standard deviation of the onset to treatment time is taken as 120 minutes, based upon available national audit data.^11^

Treatment rate: Assuming a conservative baseline thrombectomy rate of 3% in the total stroke population across centres, an important absolute change in procedures would be 2% (ie, a decrease to 1% or increase to 5%), which is equivalent to a change of 8.4% amongst the primary analysis population (baseline 13.2%, decrease to 4.8% or increase to 21.6%). To confirm whether the intervention changes treatment rates compared with standard care, *894* participants are required to detect an 8.4% difference (using change from 13.2% to 21.6% which gives the higher sample size, two-sided 5% significance level; power 90%; ICC 0.01 for 150 clusters with 1:1 randomisation).

The total size of the primary analysis population, however, will be dependent on CSC thrombectomy rates as this will determine the availability of patients for the time to treatment analysis. Based on previously published work,^11 21^ the investigator team have estimated that the primary analysis population size will be between 2600 and 4300 patients if thrombectomy services are running at 5% and 3% of all stroke patients, respectively.

The total size of the full study population is dependent on the rate at which suspected/stroke patients present to services, and the size and opening order of regions taking part in the trial. Estimations using historical data suggest that the full study population will be approximately 80 000 patients.

Because the data required for the time to thrombectomy treatment analysis are mandatory national audit fields, the sample size estimate has not been inflated for attrition. However, availability of these data will be regularly monitored and trial enrolment will not cease until appropriate data are available for 564 relevant thrombectomy treated patients. As described above, to achieve this number of thrombectomy treatments, the sample size estimate for treatment rate should be easily exceeded and will therefore allow for any potential attrition in receipt of thrombectomy information.

### Study timeline

Trial enrolment commenced in the first participating region on 7 November 2022. Enrolment will end when time to thrombectomy treatment data are available for the required 564 patients from the primary analysis population. At submission of this manuscript, five NHS ambulance services which routinely convey patients to 26 PSCs and eight CSCs are involved in the study. The eight CSCs receive SPEEDY pathway calls from regional ambulance practitioners randomised to the intervention group. Accruing study data are regularly monitored and relevant time to thrombectomy treatment information for 564 participants in the primary analysis population is expected to be obtained by December 2025. First study results are expected in summer 2026.

### Statistical analyses

All analyses and analysis populations will be fully described in a detailed statistical analysis plan.

#### Primary analysis

The primary analysis will use the primary analysis population with the estimands framework strategy^36^ applied to intercurrent events to define the analysis groups. The intercurrent events of importance and the chosen estimands strategies are shown in table 2.

**Table 2:** Intercurrent events of importance in the SPEEDY trial

Intervention group		Control group	
Intercurrent event	Strategy	Intercurrent event	Strategy
1. SPEEDY pathway not initiated or telephone call not answered.	Principal stratum	1. SPEEDY pathway initiated and call answered	Principal stratum
2. No conveyance decision made during SPEEDY pathway call (eg, call abandoned) or decision is conveyance to local PSC due to capacity limitations at the CSC.	Treatment policy		

CSC, comprehensive stroke centre; PSC, primary stroke centre; SPEEDY, Specialist PrE-hospital rEDirection for ischaemic stroke thrombectomY.

*Intervention primary analysis group*: this comprises only patients where the SPEEDY pathway was initiated/contact with the CSC was made. Patients where the attending intervention group assigned ambulance practitioner fails to initiate the pathway or cannot establish contact with the CSC will not feature in this analysis group. However, where the pathway is initiated and contact with the CSC is achieved, patients will remain in the group regardless of the conveyance decision made (ie, whether they are redirected to the CSC or sent to the local PSC) and whatever treatment decision is made (ie, whether they receive a thrombectomy or not via either route).

This approach to defining the intervention primary analysis group is being taken because it reflects the impact of the SPEEDY pathway when it is used during patient care and separates this from the additional impact of ambulance service deployment rate. As a cluster trial (chosen to minimise contamination), if a classic intention to treat (ITT) approach is employed, the group primary outcome data (thrombectomy rate and time from onset to treatment) become influenced by ambulance service deployment rates of a research intervention, ie, because the intervention group in this cluster trial comprises any patient attended by an intervention assigned ambulance practitioner, the group would contain both patients who had received the pathway and those where the attending practitioner had not deployed it.

As a research project, it is not possible to take advantage of systems used to enforce clinically adopted pathways across a large dispersed ambulance workforce such as mandatory audit, staff training and regular feedback. Instead, research relies on softer approaches for engagement such as regular communication and attempting to maintain a positive research culture. Ambulance assessed patients meeting the pathway criteria do not occur frequently for individual practitioners. It is estimated that an average of 1–2 suspected stroke patients per month are assessed by an individual practitioner, but only every 4–6 months will one of these patients fulfil the pathway initiation criteria. Therefore, individual practitioners are required to recognise an infrequent event under time pressure in the uncontrolled prehospital environment, and then remember to deploy a research intervention.

Using this described estimands intercurrent events strategy to define the intervention primary analysis group allows the impact of the pathway to be assessed without influence of an unpredictable deployment rate. This approach also makes the results more generalisable to any healthcare setting (inside and outside the UK) where any future deployment (implementation) rate may differ for different reasons.

*Control primary analysis group*: this comprises only patients where the SPEEDY pathway was NOT initiated. Any patients where the SPEEDY pathway was used will be excluded. This scenario, however, where the pathway is initiated by control assigned staff (in error), should be infrequent due to the cluster design.

*Analysis*: using the estimands defined groups, the co-primary outcomes of thrombectomy rate and time to thrombectomy will be examined using a two-part regression model. The model will be adjusted for predefined baseline covariates and the stratification variables of size of ambulance station and distance from a CSC.

Part 1 of the model will determine the chance of thrombectomy treatment occurring as a binary variable. Part 2 of the model will then analyse time to treatment as a continuous variable, conditional on thrombectomy having occurred.

Hypothesis testing for the co-primary outcomes will use a fixed-sequence procedure to account for multiple testing.^37^ The hypothesis for time from symptom onset to thrombectomy will be tested first at a 5% significance level. Only if the null hypothesis for time to treatment is rejected will the hypothesis for thrombectomy rate be tested, also at a 5% significance level. This sequence (ie, time before rate) is chosen because the effect of the trial intervention (SPEEDY pathway) on time to treatment is more important clinically than an effect on rate.

To conclude that the SPEEDY pathway is effective, a statistically significant difference between the groups is required for time to thrombectomy, *or*, both time to thrombectomy and thrombectomy rate.

As described above in ‘Sample size’, there should be no missing data for the co-primary outcomes and therefore an imputation strategy should not be required.

#### Secondary analyses

Using the primary analysis population, a standard ITT approach will be applied to examine the rate and speed of thrombectomy treatment.

Secondary outcomes will be evaluated using both the primary and other analysis populations. According to the type of data, linear or generalised linear models will be used. Both unadjusted analyses and analyses which adjust for predefined baseline covariates and the stratification variables will be undertaken. Analyses will be on a complete case basis, although if required, additional sensitivity analyses will be undertaken using an appropriate imputation method.

#### Performance analyses

The performance of the study intervention pathway for identification of patients suitable for thrombectomy will be determined by calculation of sensitivity, specificity, positive predictive value and negative predictive value.

### Economic evaluation

The economic evaluation will include within-trial cost-effectiveness analyses, cost–utility analyses and calculation of willingness to pay at a range of accepted threshold values. Due to the different study populations contained within the trial, several separate evaluations may be conducted which will be fully detailed in a Health Economic Analysis Plan. The perspective will be that of the English National Health Service and all assumptions will be stated and justified by relevant source data.

For an initial set of cost-effectiveness analyses, incremental effects will be measured in terms of the volume of patients treated and difference in minutes until the start of the thrombectomy procedure for treated patients. Recorded resource usage will enable incremental costs to be calculated using standard reference values for ambulance practitioner and remote specialist time, ambulance journeys, thrombectomy procedures, thrombolysis and hospital bed usage according to diagnostic category. The cost per thrombectomy and per minute of reduction in time until treatment will be reported. A sensitivity analysis will examine the effect of including resource utilisation due to patients who did not receive thrombectomy but underwent a remote assessment and/or were transferred to a CSC.

Further cost-effectiveness analyses will be conducted where effectiveness will be measured in terms of modified Rankin Score at the end of the care episode, ie, final discharge into the community (±local repatriation) or death, to report incremental cost per patient dependency level including death. As it is possible that the specialist prehospital redirection intervention could lead to a relative increase in treatments late within the clinical time window that reduce the overall mean benefit for health, further analysis will specifically examine the cost per independent patient stratified according to the time interval between stroke onset and thrombectomy treatment.

Cost–utility analyses will estimate lifetime incremental cost per quality-adjusted life-year (QALY), following the National Institute for Health and Care Reference Case.^38^ Data from the trial indicative of health status (specifically arterial reperfusion according to TICI score,^39^ clinical recovery according to NIHSS score at 24hours and mRS at discharge)^40 41^ will be combined with reference data about longer term stroke outcomes (quality of life and healthcare utilisation) and lifetables to estimate the lifetime marginal cost utility of the pathway. Data will be incorporated into a Markov model to determine the relative clinical and resource efficiency of the intervention, measured in terms of projected incremental cost per QALY. A cost-effectiveness acceptability curve will be generated using bootstrapped estimates of incremental costs and QALYs to illustrate uncertainty surrounding the cost-effectiveness estimate. The probability that the intervention would be considered cost-effective according to a range of willingness to pay values will also be calculated.

### Process evaluation

A two-part parallel process evaluation is being conducted to explore professional and patient/family member views and experiences about the novel specialist prehospital redirection thrombectomy pathway. Part 1 occurred during the first few months of the trial which were considered an internal pilot study as required by the funder of the project. Part 2 occurs during the remainder of the trial.

During part 1, a rapid cycle of semistructured interviews with individual ambulance and hospital practitioners (n=10 to 20) sought initial experiences and views regarding fidelity and logistical challenges related to the intervention pathway. Relevant staff involved in pathway delivery were contemporaneously identified at participating NHS organisations and invited to take part. Following provision of information and consent, online (MS Teams or Zoom) or telephone interviews were conducted with a university-based qualitative researcher. An interview topic guide was used to facilitate discussion, and data collection and analysis occurred concurrently to allow for issues or themes identified in earlier interviews to be explored in more depth in subsequent interviews. All interviews were digitally recorded and transcribed verbatim. Analysis was based on the Framework approach^42^ using a mainly deductive and pragmatic qualitative health research approach.

Part 2 involves interviews with both healthcare professionals (approximately 20) and patients with or without a family member (approximately 20) to seek views and experiences about the intervention pathway.

#### Healthcare professionals

Ambulance practitioners and CSC staff who have been involved in using the intervention pathway in each region are identified at participating NHS organisations and invited to take part. Regional involvement is staggered for logistical purposes. Following provision of information and consent, online (MS Teams or Zoom) or telephone interviews are conducted which last approximately 30 minutes. As described above, university-based qualitative researchers conduct the interviews facilitated by a topic guide, all are recorded and transcribed with analysis based on the Framework approach.^42^

#### Patient and family members

Patients from each trial region who experience the intervention pathway and are redirected to a CSC with or without subsequently receiving thrombectomy are eligible to take part in the interviews provided that they have mental capacity to provide consent and are medically stable. Any family member aware of the patient’s situation is also eligible to be interviewed to create a patient/family member dyad. Interviews can be held separately or together according to the patient/family member dyad preference. Patients who do not have an appropriate family member or a family member does not wish to participate, can still take part alone. To facilitate recall of pathway and care details, interviews are intended to be held as soon as is practically possible after emergency care processes have been completed.

Eligible patients/family members are identified and approached at participating CSCs. Following provision of an information sheet and time to consider participation, those willing are asked to provide consent for their contact details to be given to the university-based qualitative researcher. The qualitative researcher will subsequently make contact, answer any further questions and make the logistical arrangements for the interview (eg, date, time, preference for online or telephone appointment). Consent will be obtained prior to the interview which will be facilitated by a topic guide, recorded and transcribed, and analysis based on the Framework approach.^42^

## Patient and public involvement

This research project has a public representative as an investigator and an additional public representative on the independent Trial Steering Committee. Furthermore, a project-specific ‘public partnership group’ was established to provide input and advise on all aspects of the study. This group comprises 12 individuals from across England who are either stroke survivors or carers.

## Ethics and dissemination

Ethical approval for this study was obtained from the North East—Newcastle & North Tyneside 1 Research Ethics Committee (reference: 22/NE/0103). Health Research Authority and participating NHS Trust approvals are also in place.

Dissemination of study results will include presentations at national and international conferences and events, publication in peer-reviewed journals, and plain English summaries for patient/public engagement activities.

## Data availability statement

The datasets generated or analysed in this study are not expected to be made available due to governance regulations.

## Discussion

Thrombectomy for LVO stroke is clinically and cost effective but access to treatment is problematic across the UK and in many other international healthcare settings.^10 12^ The SPEEDY cluster RCT is evaluating a specialist redirection pathway where people with likely LVO stroke who are potentially suitable for thrombectomy are identified during prehospital emergency assessment and selectively redirected to the regional CSC.

The pathway design aims to support identification of appropriate patients and easily integrate into existing healthcare infrastructure, such that it could be rapidly adopted across the NHS if beneficial. The pathway has been limited to patients who are within 5 hours of symptom onset or within 5 hours of waking up with symptoms to reflect the main clinically eligible thrombectomy population. Although more recent clinical trials have reported that thrombectomy is effective up to 24 hours since patients are last known to be symptom free,^27^ selection of such cases for treatment requires advanced brain imaging which is not yet part of standard care in some PSCs.

Due to the nature of the pathway intervention, a cluster trial design was chosen to minimise group contamination, and a waiver of consent granted without which the study would have been impractical to deliver and/or have considerable bias. Although the pathway is only intended for use with some suspected stroke patients, enrolment to the trial comprises all patients with suspected and confirmed stroke across participating regions. This approach allows the impact of the pathway to be evaluated within several analysis populations including the primary analysis population where direct effects of the intervention are expected, but also other populations where indirect effects may be observed. Data for the study are being obtained from routine healthcare records and while this improves efficiency, it may also raise concerns about data quality.

The study has parallel health economic and process evaluations, which add value and provide additional information about the impact of the SPEEDY pathway.

## Supplementary material

10.1136/bmjopen-2025-112545 online supplemental file 1
