# Supplementary material for: Specialist PrE-hospital rEDirection for ischaemic stroke thrombectomY (SPEEDY): study protocol for a cluster randomised controlled trial with included health economic and process evaluations
Source: BMJ Open. 2026 Jan 13;16(1):e112545. doi: 10.1136/bmjopen-2025-112545 (PMC12815104; doi:10.1136/bmjopen-2025-112545)
Supplement: online supplemental file 1 [file bmjopen-16-1-s001.docx]

**Specialist PrE-hospital rEDirection for ischaemic stroke thrombectomY (SPEEDY): study protocol for a cluster randomised controlled trial with included health economic and process evaluations**

**Supplementary file: Data items obtained in each dataset**

**1. Ambulance assessment parameters when stroke was suspected by ambulance practitioners: from routine ambulance care records**

Attending ambulance practitioner ID numbers (likely 2 practitioners per incident) or station/team allocation

Ambulance incident/computer dispatch number

Age

Month and year of date of birth

Gender

FAST test result

Other symptom(s) suggesting acute stroke

Seizure (yes/no)

Headache (yes/no)

Past history of epilepsy (yes/no)

Past history of migraine (yes/no)

Glasgow Coma Scale (GCS) score

Alert, Voice, Pain, Unresponsive (AVPU) scale score

First blood pressure reading

First body temperature reading

First peripheral oxygen saturation reading

First heart rate reading

Capillary blood glucose reading

Suspected stroke symptom onset or last know well date and time

Care process key time intervals (date/time of: 999 call, ambulance dispatch, scene arrival, scene departure, hospital arrival)

Destination hospital (i.e. first admission hospital)

Free text details about any use of the study intervention pathway

Secondary transfer to a study CSC (yes/no) with departure and arrival date/time

**2. Hospital clinical characteristics, care received and diagnostic category for all stroke patients: from SSNAP data**

First admission hospital

First admission hospital arrival date/time

Month and year of date of birth

Age

Gender

SSNAP record identifier

First ambulance incident/computer dispatch number

Diagnosis categorised as: ischaemic stroke, haemorrhagic stroke.

If ischaemic stroke, was this due to large vessel occlusion (yes/no)

If large vessel occlusion how was this determined (clinically/from imaging)

If large vessel occlusion, where was the occlusion (anterior circulation / posterior circulation)

If haemorrhagic stroke, diameter of haematoma on first brain imaging

Symptom onset or last known to be well date/time (hospital specialist judgement)

Wake up symptoms (yes/no)

Stroke symptom severity on admission (National Institute of Health Stroke Score (NIHSS) including all component scores)

First blood glucose reading on admission (capillary or serum glucose)

First blood pressure reading on admission

First heart rate reading on admission

First body temperature reading on admission

First peripheral oxygen saturation on admission

Past history of Atrial Fibrillation (yes/no)

New Atrial Fibrillation on this admission (yes/no)

On anticoagulant medication prior to admission (no; vitamin K antagonists; DOAC; heparin)

Pre-stroke dependency (Modified Rankin Scale score)

Modality(ies) of first brain imaging (Plain/non-contrast CT; CT Intracranial angiogram; CT Perfusion; Plain/non-contrast MRI; Contrast-enhanced MRA; MR Perfusion)

Dates/times of first brain imaging

Receipt of thrombolysis (yes/no)

Date/time of thrombolysis (if received)

If thrombolysis not received reason why

Agent (drug) used for thrombolysis

BP lowering treatment given (yes/no)

If BP lowering treatment not given reason why

Anticoagulant reversal treatment given (yes/no)

If anticoagulant reversal treatment not given reason why

Stroke symptom severity at 24 hours post any reperfusion treatment (National Institute of Health Stroke Score (NIHSS) including all component scores)

Intracranial haemorrhage post any reperfusion treatment (yes/no)

Referral for thrombectomy (no, yes accepted at admission hospital team, yes accepted at another team, yes but declined)

If transferred to another hospital for thrombectomy, date/time of departure and arrival

Receipt of thrombectomy (yes/no)

If thrombectomy not received after acceptance reason why (pre-procedure imaging demonstrated reperfusion so procedure not required; pre-procedure imaging demonstrated absence of salvageable brain tissue; other)

If thrombectomy received, date/time of arterial puncture; date/time of end of procedure

Neurosurgical consultation (yes/no)

Neurosurgical transfer (yes/no)

At discharge/transfer from each hospital where care was received:

-outcome (died, discharged to care home, discharged home, discharged elsewhere, transferred to another inpatient care team).

-if discharged, date of discharge

-dependency (Modified Rankin Scale score)

**3. Thrombectomy treatment parameters (if received): ‘CSC thrombectomy treatment log’**

*(there is some overlap in data items between this log and SSNAP data to support reporting of trial progress and safety because SSNAP data can only be supplied several months in arrears)*

CSC name

CSC arrival date/time

First admission hospital name

First admission hospital arrival date/time

First ambulance incident/computer dispatch number

Month and year of date of birth

Age

Gender

SSNAP record identifier

Route to thrombectomy: transfer from PSC, direct admission to CSC. If direct admission to CSC, was this via the SPEEDY pathway

Symptom onset or last known to be well date/time (hospital specialist judgement)

Wake up symptoms (yes/no)

Stroke symptom severity on admission (National Institute of Health Stroke Score (NIHSS) including all component scores)

First blood glucose reading on admission (capillary or serum glucose)

First blood pressure reading on admission

Pre-stroke dependency (Modified Rankin Scale score

Receipt of thrombolysis (yes/no)

Date/time of thrombolysis (if received)

If thrombolysis not received reason why

Brain imaging/techniques performed before thrombectomy (CTA; MRA; measurement of ASPECTS score; assessment of ischaemic penumbra via CT/MR/both)

Anaesthesia during procedure (local; general; both)

Date and time of arterial puncture

Date and time of end of procedure

Method(s) used to open culprit vessel occlusion (yes or no to: thrombo-aspiration; stent retriever; proximal balloon/flow arrest guide catheter; distal access catheter)

Procedure complications (yes or no to: distal clot migration/embolisation within affected territory; embolisation to new territory; intracerebral haemorrhage; subarachnoid/intraventricular haemorrhage; arterial dissection or perforation; vasopasm; other

Angiographic appearance of culprit vessel pre-procedure (modified TICI scale score)

Angiographic appearance of culprit vessel post-procedure (modified TICI scale score)

Stroke symptom severity at 24 hours post any reperfusion treatment (National Institute of Health Stroke Score (NIHSS) including all component scores)

At discharge/transfer from CSC:

-outcome (died, discharged to care home, discharged home, discharged elsewhere, transferred to another inpatient care team).

-if discharged, date of discharge

-dependency (Modified Rankin Scale score)

**4. Study intervention pathway content (if received): ‘CSC SPEEDY call log’**

CSC name

Date and time of call

Ambulance practitioner ID number

Month and year of date of birth

Age

Gender

Study pathway content: nearest stroke unit, FAST results, symptom onset time, AVPU score, other details captured during remote assessment, destination decision.

Date and time of end of call

CSC staff involved in remote assessment (yes or no to: stroke nurse; stroke nurse practitioner; trainee doctor; stroke consultant; other (if other state role)

For patients who undergo direct CSC admission via the pathway, the ‘CSC SPEEDY call log’ will also capture additional detail about the subsequent hospital assigned diagnosis and treatments received.
