# Supplementary material for: Specialist PrE-hospital rEDirection for ischaemic stroke thrombectomY (SPEEDY): study protocol for a cluster randomised controlled trial with included health economic and process evaluations
Source: BMJ Open. 2026 Jan 13;16(1):e112545. doi: 10.1136/bmjopen-2025-112545 (PMC12815104; doi:10.1136/bmjopen-2025-112545)
Supplement: Supplementary file 2 [file bmjopen-2025-112545.reviewer_comments.pdf]

PEER REVIEW HISTORY

BMJ Open publishes all reviews undertaken for accepted manuscripts. Reviewers are asked to complete a checklist review form and are provided with free text boxes to elaborate on their assessment. These free text comments are reproduced below.

ARTICLE DETAILS

Title (Provisional)

Specialist PrE-hospital rEDirection for ischaemic stroke thrombectomY (SPEEDY): study protocol for a cluster randomised controlled trial with included health economic and process evaluations

Authors

Shaw, Lisa; Allen, Michael; Day, Jo; Ford, Gary; James, Martin; McClelland, Graham; Mcmeekin, Peter; Mossop, Helen; Pope, Catherine J; Simmonds, Rosemary L; White, Phil; Wilson, Nina; Price, Christopher

---

This article was not externally reviewed at BMJ Open. Protocols that have been independently assessed prior to submission to BMJ Open are usually fast-tracked to publication on the grounds that further substantial changes will not be possible. This independent assessment will usually be external, independent review for both a substantial grant award from a non-commercial or government funder and ethics approval.
